# 1333. A National Outbreak of Respiratory Syncytial Virus Associated with Emergence of a Genetically Distinct ON1 variant

**DOI:** 10.1093/ofid/ofab466.1525

**Published:** 2021-12-04

**Authors:** Wei Hsuan Lin, Fang-Tzy Wu, Yi-Yin Chen, Kuo Chien Tsao, Yu-Chia Hsieh

**Affiliations:** 1 Department of Pediatrics, Chang Gung Children’s Hospital, Chang Gung Memorial Hospital, Chang Gung University, College of Medicine, Taoyuan, Taiwan, Guishan, Taoyuan, Taiwan; 2 Department of Health, Research and Diagnostic Center, Centers for Disease Control, Taiwan, Guishan, Taoyuan, Taiwan; 3 Linkou Chang Gung Memorial Hospital, Taoyuan City, Taoyuan, Taiwan (Republic of China); 4 Research Center for Emerging Viral Infections, Chang Gung University, Taoyuan, Taiwan, Guishan, Taoyuan, Taiwan

## Abstract

**Background:**

A national outbreak of respiratory syncytial virus (RSV) has been observed in the community since Fall 2020 in Taiwan.

**Methods:**

We reviewed a national laboratory-based surveillance network established over 20 years by Taiwan Centers for Disease Control (TCDC) for respiratory viral positivity rate and viral pathogen in outpatient department and hospitalized patients. A retrospective study of children younger than 5 years old hospitalized with RSV infections at Chang Gung Memorial Hospital (CGMH) including Lin Kou and Kaohsiung branch between 2018 and 2020 was conducted. Samples positive for RSV A were sequenced. Patients’ clinical data was obtained from medical files and stratified by genotype and year.

**Results:**

2020 showed a 4-fold surge in RSV cases in Taiwan, in which surpassed both 2011 (the appearance of ON1 strain in Taiwan) and 2013 (ON1 strain predominates in Taiwan)(Figure1,Table1). Phylogenetic analysis of G protein sequences showed that most strains in 2020 were clustered apart from 2018, 2019 seasons and other ON1 reference strains between 2011 and 2016, indicating a novel ON1 variant had been circulating in the community(Figure2). The novel ON1 variants carried six amino acid changes, of which T113I,V131D, H258Q,H266L,Y304H located in the mucin domains and N178G in central conserved domain. These changes emerged gradually in 2019 and showed a high consistency in 2020. The unique amino acid substitution E257K in the second mucin domain was noticed in 2020 exclusively. Besides, 10 substitutes in F protein were appeared between 2018 and 2020, of which R213S and N276S were in antigenic sites. Furthermore, substitutes T12I and H514N in F protein were first emerged since 2020. On multivariate analysis, age (OR 0.97; 95% CI 0.94-0.99; p 0.02) and ON1 variant in 2020 (OR 2.52; 95% CI 1.13-5.63; p 0.025) were independently associated with oxygen saturation < 94% during hospitalization (Table 2).

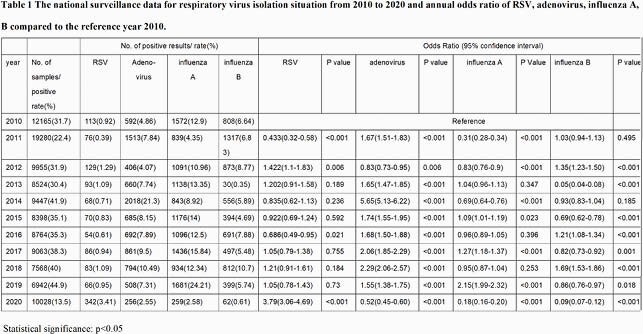

Figure 2. The phylogenetic tree of G protein sequences including ON1 strains in our study from 2018 to 2020 and reference strains from 2011 to 2016

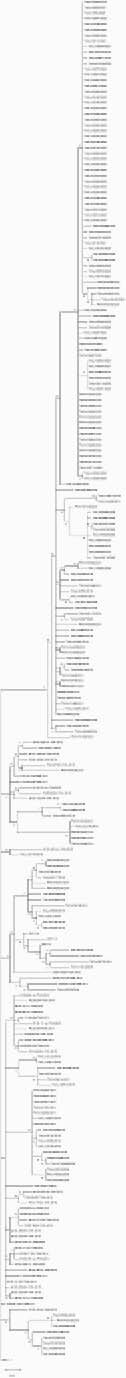

The phylogenetic tree showed that most strains of the 2020 season clustered apart from those of the previous seasons, which indicated there was a novel ON1 variant circulating in the community associated with 2020 RSV epidemic.

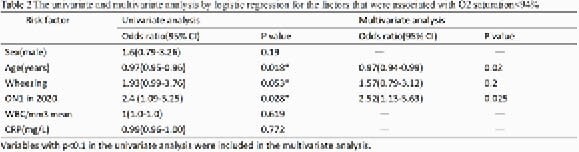

Variables with p<0.1 in the univariate analysis were included in the multivariate analysis.

**Conclusion:**

An unprecedented RSV epidemic within the last 10 years caused by a novel ON1 variant has occurred in 2020 (Figure 1), suggesting the sets of mutations may confer fitness advantage. Further studies on viral replication, infectivity and virulence is needed to understand the evolution and spread of RSV.

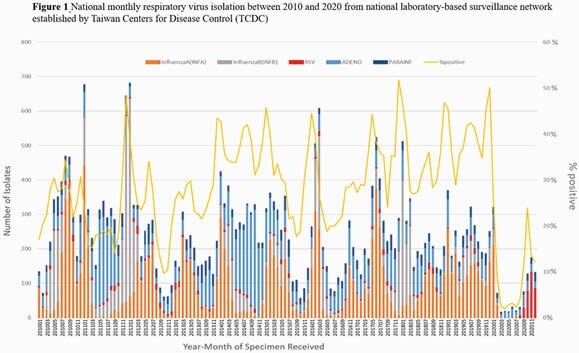

**Disclosures:**

**All Authors**: No reported disclosures

